# Gastrointestinal Parasitism in Miranda Donkeys: Epidemiology and Selective Control of Strongyles Infection in the Northeast of Portugal

**DOI:** 10.3390/ani11010155

**Published:** 2021-01-11

**Authors:** Sérgio Ramalho Sousa, Sofia Anastácio, Miguel Nóvoa, Adolfo Paz-Silva, Luís Manuel Madeira de Carvalho

**Affiliations:** 1 Vasco da Gama Research Centre (CIVG), Department of Veterinary Sciences, Vasco da Gama University School, Avenida José R. Sousa Fernandes 197 Lordemão, 3020-210 Coimbra, Portugal; ramalhosousa@gmail.com (S.R.S.); sferreira.anastacio@gmail.com (S.A.); 2 CIISA—Centro de Investigação Interdisciplinar em Sanidade Animal, Faculdade de Medicina Veterinária, Universidade de Lisboa, Avenida da Universidade Técnica, 1300-477 Lisbon, Portugal; 3 Centre of Neuroscience and Cell Biology (CNC), University of Coimbra, 3004-531 Coimbra, Portugal; 4 Association for the Study and Protection of Donkeys (AEPGA), Largo da Igreja no. 48, 5225-011 Miranda do Douro, Portugal; miguelnovoa@aepga.pt; 5 Control of Parasites Group (COPAR, GI-2120), Department of Animal Pathology, Veterinary Faculty, University of Santiago de Compostela, 27002 Lugo, Spain; adolfo.paz@usc.es

**Keywords:** Miranda donkeys, gastrointestinal parasites, epidemiology, Strongylidae, Cyathostominae, targeted selective treatment, Portugal

## Abstract

**Simple Summary:**

In the northeast of Portugal, the Association for the Study and Protection of Donkeys (AEPGA) works to recuperate the autochthonous Miranda do Douro donkey breed. Donkeys living in the three rehabilitation centers of the association have been assessed since 2005 with regards to the parasite control, since parasitism is an important issue affecting their health. Firstly, a strategic approach was implemented for parasite control, but over the years, a lack of anthelmintic efficacy over cyathostomins was observed. Thus, a different strategy was designed, using a targeted selective treatment (TST), and the effects of this approach were studied through the evaluation of parasite dynamics in the animals. Generally, it was observed that TST promoted a rational use of resources, as well as a reduction of parasite prevalence, although with a reduction of parasite biodiversity.

**Abstract:**

In Portugal, equine parasitism in pasture animals is characterized by high parasitic burden and high helminthic biodiversity; both factors are potentially pathogenic for their hosts. The decrease in the number of donkeys over the last years in Portugal, their importance in rural lowland and mountain ecosystems and pastures and the scarce information regarding their parasitism led to this research, which aimed to evaluate the parasitological status of a Miranda donkey breed population, a native breed mainly located in the northeast of Portugal. This study provides better knowledge of their gastrointestinal parasitism, particularly strongyles, and the assessment of a targeted selective treatment (TST) as an alternative control approach of their parasitism. A longitudinal observational study was developed during a period of five years in a population of 62 Miranda donkeys. At first, strategic deworming of these animals was performed every semester, but this was progressively replaced by a TST approach according to the levels of Eggs per Gram (EPG). This new deworming strategy was conducted in association with a regular parasitological monitoring of the animals every three months, being dewormed with ivermectin when egg shedding was higher than 500 EPG. Over the study period, a decrease of the annual prevalence rate of infection by gastrointestinal strongyles was observed, from 35.5% to 19.4%, as well as a negative binomial distribution of parasitic strongyles in donkeys submitted to selective anthelminthic control. The prevalence rate of infection was higher in females (39.5%), in individuals under four years (46.7%) and in those presenting a lower body condition (40.8%). The egg output was higher in animals younger than four years (589.3 EPG) than in those older than 10 years (533.6 EPG) (*p* < 0.05). However, no differences were observed according to sex during the study period. Results from this study allowed to note the influence of swampy pastures and of the weather changes in the epidemiology of strongylosis in Miranda donkeys. Moreover, it was possible to establish the annual epidemiological curve of strongyle egg shedding, with June being the month with the highest EPG, December having the lowest EPG and March and September showing intermediate numbers. Overall, a lower biodiversity of gastrointestinal parasites was observed. *Cyathostomum* sensu lato was the most prevalent genus and *Strongylus vulgaris* was the most observed large strongyle of the Strongylidae family. *Trichostrongylus axei* and *Parascaris* sp. were other nematodes with a minor frequency. The higher prevalence of strongyles at the beginning of the study showed a progressive decrease throughout the research period, and also for parasite biodiversity. Therefore, a targeted selective treatment seems to be a rational anthelminthic control approach in Miranda donkey strongyle infection and in other gastrointestinal parasites, since it reduces the antiparasitic treatments, the parasite’ prevalence and the EPG level. However, a loss of parasite biodiversity was noted at the end of the study period, as Cyathostominae were the only isolated strongyles. This can be a challenging situation in the long run, taking the ability of these nematodes to adapt easily to any deworming program, meaning that fecal EPG monitoring should be kept as a rule to a rational parasite control program.

## 1. Introduction

The donkey population has experienced a strong regression all over the world since the 1970s. However, since 2010, a reversal of this trend has been observed, and in 2013, the global population of donkeys was estimated to be 43.5 million (44.5% in Africa, 38.7% in Asia, 15.5% in America and 1.2% in Europe) [1].

In Europe, the population of donkeys is found mainly in Mediterranean countries such as Portugal, Spain, France, Italy, and Greece. From 1970 to 2013, the numbers of donkeys decreased about 78% [2,3] and autochthonous breeds suffered a risk of extinction [3,4].

In Portugal, a decrease of 60% was observed from 1999 to 2009 [5]. This occurred because of the mechanization of agriculture, as well as the desertification of the countryside, with the consequent disappearance of cultural and traditional values [6]. However, between 2010 and 2013, an increase of 5000 animals was observed [1]. A renewed interest in donkeys was recorded in many countries, including Portugal, where a recovery program of the unique autochthonous breed of donkeys in the northeast of Portugal (Miranda do Douro donkey breed) was implemented. Donkeys began to be used in therapeutic activities, leisure events, ecotourism and as companion animals [7,8].

Donkeys are known by their strength and robustness. However, they are vulnerable to parasitic diseases [9,10], and helminthic parasitism is recognized as the main cause of morbidity and mortality in these animals [10,11,12]. Weakness is a common sign in donkeys infected by strongylids and cyathostomins. Nevertheless, these animals are characterized by a high resistance and resilience. Frequently, the occurrence of mixed infections, sometimes by more than 20 different species of strongylids per host, are reported [13,14,15,16].

Studies focusing parasitism on donkeys are limited and, consequently, the data about parasite biodiversity, prevalence, incidence and effects on the animal health are scarce [12,17]. However, the implementation of appropriate therapeutical and/or prophylactic measures regarding donkey parasites at population level depends on this knowledge [18,19]. It is known that internal parasitism associated with hard work, stress and malnutrition are causes of death in donkeys [12]. Thus, the nonexistence of the sustainable control of parasites increases the negative impact of parasitism in donkeys [20].

Therefore, the prevention and control of parasitic infection is particularly important in donkeys to improve animal health, which influences several factors, such as the reproduction ability [11,21,22,23].

Interestingly, from the available data, the infection by strongylids in donkeys is common in regions showing diverse geographical and climatic characteristics and the dominant species of parasites seem to be similar in different regions. Several authors refer simultaneously to high levels of infection, prevalence and biodiversity [12,24,25].

In Portugal, in grazing equids, individual parasitism is characterized by a high parasite infection level and by high parasite biodiversity, including highly pathogenic species [26,27,28]. 

Knowledge about parasite biodiversity, associated with the use of adequate anthelminthic drugs in the most suitable seasons, with the usage of the right forages as natural dewormers, are important tools to provide an integrated and effective control of parasite infection in donkeys [26,27].

A strategic deworming approach was conducted, from 2005 to 2008, in donkeys from the autochthonous breed in the northeast of Portugal (Miranda do Douro donkey breed). This methodology followed a defined schedule of anthelmintic treatments in periods known for displaying a higher level of parasitism and environmental contamination. A decrease of the prevalence of gastrointestinal nematodes infection and of the biodiversity of nematodes was observed. However, the prevalence of infection by cyathostomins increased [28,29]. These findings together with those reported in other studies showed that the strategic anthelmintic control leads to a lack of efficacy regarding cyathostomins [30]. Given the importance of the anthelmintic control to impair the negative effect of parasitism on animal welfare, animal health and reproductive ability, which are very important for the recovery of the breed [6,8], a different approach was considered for anthelmintic control in the above-mentioned population of donkeys. This approach was based on Targeted Selective Treatment (TST), which consists of selective anthelmintic treatment of animals showing a medium-high level of infection (≥500 eggs per gram—EPG) [31]. Thus, the present study was designed to evaluate the effect of TST in parasitism in the population of the Miranda do Douro donkey breed. Specifically, this study aimed to analyze the dynamics of parasitism in Miranda do Douro donkeys submitted to a TST, which was associated with regular monitoring of fecal egg counts (FEC) and fecal cultures.

## 2. Materials and Methods

### 2.1. Study Design

**Figure 1:**
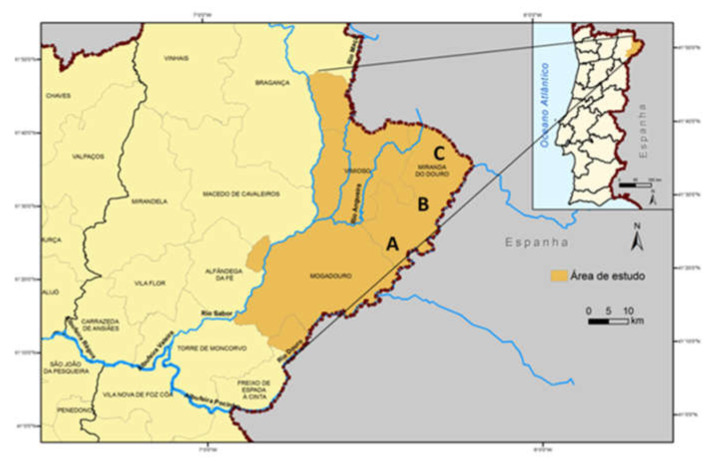
Location of the study area in the northeast of Portugal and of the rehabilitation centers of the Association for the Study and Protection of Donkeys (AEPGA) A—Atenor; B—Duas Igrejas; C—Pena Branca (adapted from Meirinhos, 201 [33]).

Between December 2009 and September 2014, an observational longitudinal study was conducted on the total population of donkeys (*Equus asinus*) living at the three Miranda do Douro donkey breed rehabilitation centers of the Association for the Study and Protection of Donkeys (AEPGA). The population of donkeys from Miranda do Douro rehabilitation centers is a nucleus of animals used in activities to preserve the breed. The animals remain in a semi-extensive production system with permanent access to pasture. Moreover, they are frequently moved between the three centers as well as outside the centers, sharing spaces and pastures with other equids, ruminants, and even other domestic animals from traditional farms in the region. The rehabilitation centers are located in three villages at the highlands in the northeast of Portugal: Atenor (41°42′ N 06°48′ W, 652 m of altitude), Duas Igrejas (41°47′ N 06°35′ W, 738 m of altitude) and Pena Branca (41°54′ N 06°26′ W, 741 m of altitude) (Figure 1).

At the beginning of the study, individual data of the animals were registered, namely identification, sex, age, body condition and thoracic perimeter. The parameters used to characterize the population followed that described in the literature and a categorization was used for analysis purposes [32]. Concerning age, three categories were considered: young (<4 years), adult (from 4 to 10 years) and old (>10 years). Regarding body condition, a scale of classification from 1 to 5 was used and three groups were considered: 2–2.5, 3–3.5 and 4–5. The thoracic perimeter allowed the estimation of the height and the weight of the animals, and three groups were considered: 115–129, 130–149 and 150–168 cm [32]. Besides individual information, data about the animal management at farm level were registered, as well as meteorological data during the study period.

From December 2009 to September 2014, the anthelmintic control in animals was based on a TST method and only those animals showing an egg output ≥500 EPG received anthelmintic treatment [31]. This means that all the animals were monitored every three months and anthelmintic treatment by subcutaneous administration of 2% ivermectin (1 mL/50 Kg body weight) was performed on animals indicated for treatment.

For 60 months, on a trimestral basis, fecal samples (500 g) were collected by rectal palpation from all the animals. Samples were stored at 5 ± 3 °C until analysis at the laboratory. Quantitative coprological assays were performed using a modified McMaster method [34]. The obtained results, eggs per gram of feces (EPG), allowed the calculation of the prevalence of strongyle infection, considering that a negative result corresponded to less than 50 EPG. According to the EPG value, it was also possible to rank the level of parasite infection (LPI) as weak LPI (50 to 450 EPG), medium LPI (500 to 1000 EPG) and strong LPI (>1000 EPG) [35,36]. Thus, deworming was performed in animals showing a medium or strong LPI, as mentioned above.

To evaluate the biodiversity of parasites, all the samples were submitted to fecal culture to obtain third-stage larvae. Fecal culture was performed following the method of Roberts and O’Sullivan modified by Ueno and Gutierres [31,35,37,38]. The morphological identification of third-stage larvae was performed following several authors, allowing the identification of genera and species based on strongylids’ L3 [31,39,40,41,42,43,44].

### 2.2. Statistical Analysis

For statistical purposes, the animal’s characterization variables were the sex (categorical nominal: female, male and gelding), age (continuous and categorical nominal: <4 years, from 4 to 10 years and >10 years), body condition (continuous and categorical nominal: 2–2.5, 3–3.5 and 4–5) and thoracic perimeter (continuous and categorial nominal: 115–129, 130–149 and 150–168 cm). Environmental characterization variables were the average temperature (continuous), minimum temperature (continuous), maximal temperature (continuous) and precipitation (continuous). Response variables were qualitative results of coprology (categorical nominal: positive/negative), EPG numbers (continuous), LPI (categorical nominal: weak, medium, strong) and number of third-stage larvae (continuous).

Data were analyzed using the statistical program “R” (version 3.2.2: https://www.r-project.org). A Spearman test was used to evaluate correlations between variables and a Kruskal–Wallis test was used to assess the variability between two or more groups. For both tests, values were considered significant for *p* < 0.05.

## 3. Results

### 3.1. Climate

Table 1 summarizes the monthly data of the weather conditions in the region during the study period. The temperature was higher than 25 °C on 401 days (22.7%), and higher than 30 °C on 284 days (16.1%). The hottest days occurred in July and August. December, January and February were the coldest months. However, in February, a higher percentage of days presented temperatures above 0.0 °C (59.5%, 55/93).

Regarding the rainfall, December, January and February were the rainiest months, and July and August were the driest months.

### 3.2. Donkey Parameters

The studied population was composed of a total of 62 donkeys, of which 42 were females (67.7%), 19 geldings (30.7%) and 1 breeding male (1.6%). Table 2 and Table 3 summarize the characteristics of the population.

**Figure 2:**
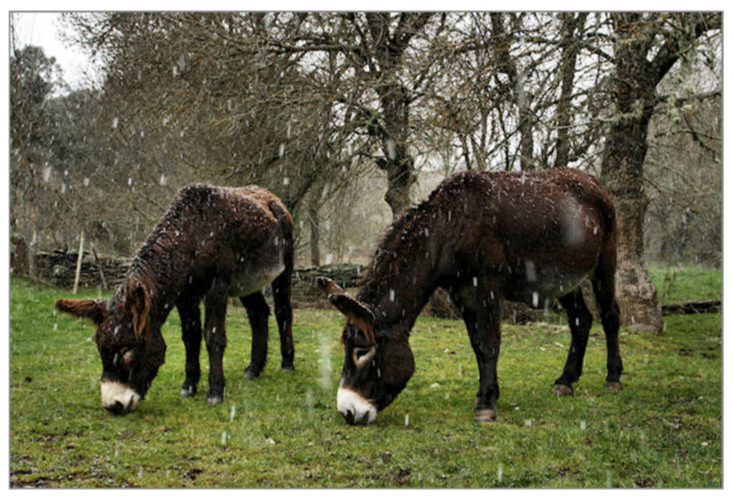
Morphological characteristics of the autochthonous Miranda do Douro Donkey breed. Grazing animals at a rehabilitation center (Source: AEPGA).

A high morphometric uniformity was observed among the studied population of the Miranda donkey breed (Figure 2).

### 3.3. Prevalence of Parasitism

**Figure 3:**
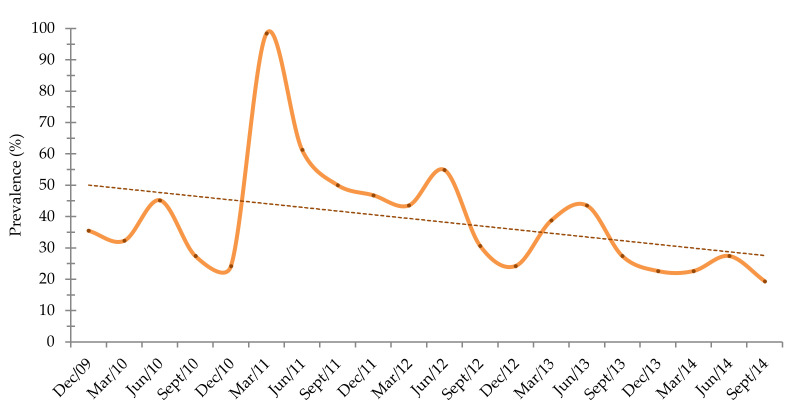
Parasitism prevalence from2009 to 2014 and linear trend line.

Over the five years, a decrease in the annual prevalence of parasitism was observed from 35.5% to 19.4% in the population submitted to a selective anthelmintic control (Figure 3). However, in the second year, an increase of the annual prevalence of parasitism was observed, and in March 2011, the prevalence of parasitism was established as 98.4%.

Overall, a seasonal variation of the prevalence was observed (Figure 3) with the higher values in March and June, and lower values in September and December.

Table 4 summarizes the average of the prevalence considering the parameters of the population. Regarding age groups, a higher prevalence was observed in animals younger than four years (*p* < 0.003).

Regarding sex, no significant differences in prevalence were observed; however, considering the number of reinfections, statistically significant results were found between females (average = 6.9, SD = 5.5) and geldings (average = 2.6, SD = 2.3) (*p* < 0.003).

It was also observed that the prevalence was higher in animals with a lower body condition index.

### 3.4. Parasitic Levels

**Figure 4:**
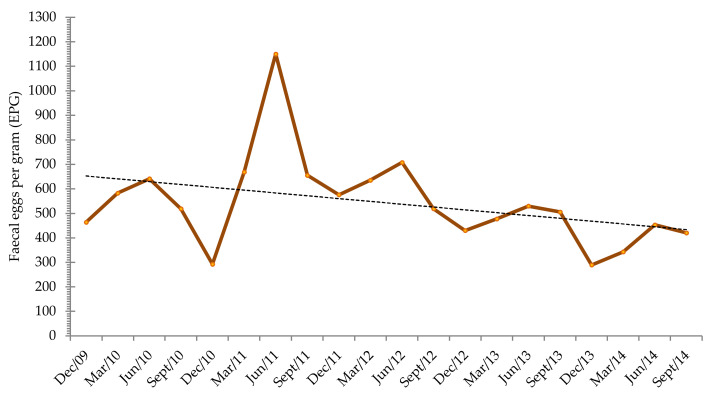
Monthly average of strongyles’ EPG during 2009–2014 and linear trend line.

Throughout the study, the mean values of EPG showed an oscillatory pattern, but a progressive reduction in the level of fluctuation was observed (Figure 4). In detail, firstly, between December 2009 and June 2011, an increase was observed; however, a progressive decrease occurred from then until September 2014. Overall, the parasitic egg shedding lessened from the first year (551.4 EPG) until the last year of the study (376.5 EPG). However, during the second and the third years, the mean values were 691.4 EPG and 609.2 EPG, respectively.

The Kruskal–Wallis test showed significant differences in the parasite level in June 2010 and June 2011 (*p* < 0.005), December 2010 and December 2011 (*p* < 0.01) and June 2011 and June 2012 (*p* < 0.005). Furthermore, significant differences were observed between the second and the fifth year of the study (*p* < 0.05) regarding egg output levels.

**Figure 5:**
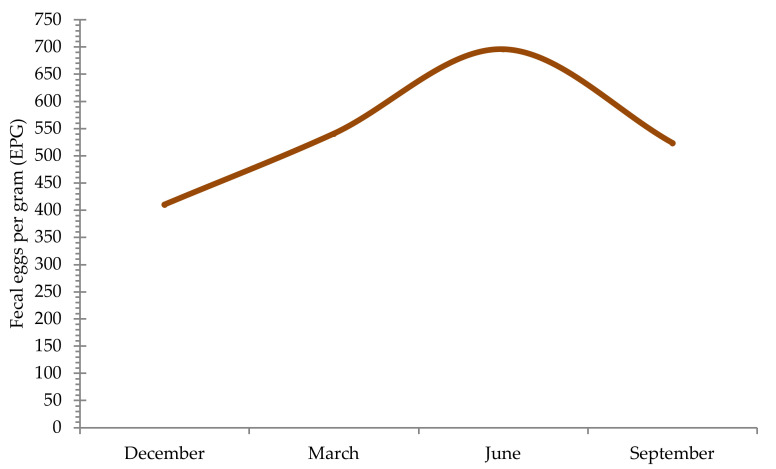
Annual average curve of Strongylid egg excretion between 2009 and 2014 in autochthonous Miranda do Douro donkeys in the northeast of Portugal.

As stated in the prevalence, a seasonality was observed in the egg excretion. In fact, in general, a higher shedding average value was observed in June (696.1 EPG), and a decrease was observed in September (523.7 EPG) and in December (410.3 EPG). From then, an increase occurred in March (541.1 EPG) until June. Briefly, an increase in the shedding of eggs occurred from December to June when a maximum was registered, and from then until next December, a progressive reduction occurred. These results allowed the establishment of the average annual excretion curve of Strongylidae eggs in Miranda do Douro donkeys in the northeast of Portugal (Figure 5).

The Kruskal–Wallis test showed that the egg output was significantly higher in animals younger than 4 years (589.3 EPG) than in those older than 10 years (533.6 EPG) (*p* < 0.05). No differences in egg output were observed according to the sex during the study period. However, over the second and the third year, the egg shedding was higher in females (779.9 and 660.1 EPG, respectively) than in geldings (554.5 and 502.3 EPG, respectively) (*p* < 0.003).

Regarding the body condition index, no significant differences were observed between groups. However, over the second year, a higher egg shedding was observed in animals from the index 2–2.5 (803 EPG). Significant differences in the egg output were observed in animals with indexes 2–2.5 and 3–3.5 (*p* < 0.001) and 3–3.5 and 4–5 (*p* < 0.001).

Concerning the thoracic perimeter, a higher egg shedding was observed in animals from the group 115–129 and significant differences were observed between groups 115–129 and 130–149 (*p* < 0.005) and groups 130–149 and 150–168 (*p* < 0.05).

Considering the number of anthelmintic treatments, during the study, the average number of deworming procedures was 3.2 in females (SD = 0.7) and 2.7 (SD = 1.1) in males, with an average deworming interval of 16.2 months in females and 22.4 months in males. The average interval between deworming is different from males to females, being shorter for females (*p* < 0.05).

### 3.5. Level of Parasite Infection (LPI)

During the study period, a decrease of the LPI was observed in the population. The prevalence of animals showing a medium LPI decreased from 45.9% to 28.5% and in those showing a weak LPI, the prevalence increased from 54.1% to 71.5%. Animals reaching a high LPI (shedding more than 1000 EPG) appeared during the second year (22.1%), remained during the third year (9.5%) and disappeared in the fourth and fifth years.

Regarding the age groups, significant differences were observed between the three groups and the different LPI (*p* < 0.0001). Moreover, the LPI fluctuated considering the gender and the months of the year. Females presented a higher number of infections with LPI equal or higher than 500 EPG than males (*p* < 0.05). 

The LPI showed a significant difference between the three groups regarding the body condition index (*p* < 0.0001). A low LPI was found mainly in animals from group 2–2.5 and a high LPI occurred mainly in animals from group 4–5. Additionally, a significant difference was found regarding the LPI and the thoracic perimeter, namely the groups 115–129 cm, 130–149 cm and 150–168 cm (*p* < 0.0001). 

Considering the meteorological variables, it was found that in animals older than 10 years, a positive correlation was observed between the average LPI (500–1000 EPG) and the mean temperature (ρ = 0.49; *p* < 0.03). In animals showing a thoracic perimeter of 130–149 cm, a positive correlation was observed between the minimum temperature and the medium LPI (500–1000 EPG) (ρ = 0.44; *p* < 0.05). A negative correlation was found between the minimum temperature and the low LPI (50–450 OPG) (ρ = −0.47; *p* < 0.036). In animals showing a body condition index of 2.5, a negative correlation was found between the minimum temperature and the low LPI (50–450 EPG) (ρ = −0.46; *p* < 0.042). In this group of animals, a negative correlation was also found between the mean temperature and a low LPI (50–450 EPG) (ρ = −0.46; *p* < 0.043), between the maximum temperature and a low LPI (50–450 EPG) (ρ = −0.63; *p* < 0.003). Still in this group, animals younger than four years showed a negative correlation between precipitation and high LPI (>1000 EPG) (ρ = −0.45; *p* < 0.045).

### 3.6. Biodiversity of Parasites

Over the five years of study, using TST, a low biodiversity of gastrointestinal parasites was observed, and cyathostomins were the most prevalent.

During the second year of study, an increase in biodiversity was observed. About 2.6% of animals presented Strongylinae nematodes, *Strongylus vulgaris* was identified in 2.0% and *Oesophagodontus robustus* was identified in 0.6%. During this period, *Trichostrongylus axei* was identified in 0.8% of animals and the genus *Parascaris* was observed in 0.8% of animals.

The genus *Cyathostomum* sensu lato was the only cyathostomin observed over the five years of study, and morphotypes A (35.0 to 85.7%) and D (15.6 to 30.8%) were the most prevalent. During the second and third years of study, an increase of the biodiversity of the genus *Cyathostomum* sensu lato was observed. The morphotypes A (46.4 to 71.7%), D (20.8 to 18.5%), C (15.7 to 15.2%), G (11.0 to 12.1%) and B (8.7 to 9.1%) were the most prevalent. The morphotypes E (2.2 to 1.5%) and F (2.2 to 1.6%) were less observed. Morphotype H was identified only in the second year of study with 0.8% prevalence. 

*Cyathostomum* sensu lato morphotype A was more prevalent in animals aged between 4 and 10 years than in those older than 10 years (*p* < 0.05), and also in animals showing a body condition index between 2–2.5 than in those between 4–5 (*p* < 0.03).

Genus *Cyathostomum* sensu lato morphotype D was observed mainly in the group of animals younger than 4 years than in those older than 10 years (*p* < 0.05), as well as in animals showing a body condition index of 2–2.5 compared to 3–3.5 (*p* < 0.05), 3–3.5 compared to 4–5 (*p* < 0.05) and even 2–2.5 compared to 4–5 (*p* < 0.0001).

The study of the abundance of third-stage larvae of genera and species of gastrointestinal parasites showed a decrease in the numbers from the first to the fifth year—from 8500 to 5701 larvae per year, respectively (Table 5).

In both the second and third years, an increase in the abundance of larvae was observed. Furthermore, a variation in the number of larvae throughout the year was observed. Overall, the abundance of larvae was lower in September (9500/47,335; 20.1%) and December (9303/47,335; 19.7%) and it was higher in March (14,337/47,335; 30.3%) and June (14,195/47,335; 30.0%).

Regarding the proportional abundance of the different genera and species, *Cyathostomum* sensu lato was the most abundant (99.8%), followed by *Trichostrongylus axei* (0.19%), *Strongylus vulgaris* (0.02%) and *Oesophagodontus robustus* (0.002%) (Table 6).

The abundance of larvae in March and June was higher compared to September and December. Over the study period, it was observed that the abundance of third-stage larvae of *Cyathostomum* sensum latum morphotype D was higher in June compared to September (*p* < 0.05).

## 4. Discussion

This study aimed to analyze the effect of a TST in the parasitological status of donkeys from the Miranda do Douro donkey breed rehabilitation centers in the northeast of Portugal. TST is based on the rational use of resources, and thus, the deworming of animals is conducted when there is indication for treatment (i.e., FEC ≥ 500 EPG). This procedure was conducted for five years. 

Over the study period, the average of deworming intervals decreased, being higher in males (22.4 months) than in females (16.2 months). These findings agree with other studies that mention a reduction in the number of anthelmintic treatments using TST [45,46,47,48,49]. Additionally, a decrease in the annual prevalence from 35.5% to 19.4% was observed, with a convergence towards the prevalence of 19.9% at the end of the study (data not shown). Similar results were reported in other studies which refer to a negative binomial type distribution in which about 20% of the hosts concentrate 80% of strongylids [45,50,51]. Thus, TST was effective in the control of strongylids’ infections in donkeys, as reported in other studies conducted in equines [45,46,47,48,49,52,53].

From 2005 to 2008, strategic anthelmintic control was used in the same population of donkeys. This strategic control consisted of treatments every six months (spring and autumn). This approach led to a decrease of the prevalence of infection, from 87.0% to 38.8% [28]. The TST used in this study allowed a decrease in the average annual prevalence of infection from 35.5% to 19.4%; moreover, the number of anthelmintic treatments was reduced.

TST is influenced by external factors, such as meteorological conditions and handling animals, as well as by internal factors, such as age, sex, body condition and size of the animal. Donkeys are animals recognized by their strength and robustness. However, helminthic parasitism is recognized as the main cause of morbidity and mortality in these animals. In Europe, autochthonous breeds of donkeys are at risk of extinction. Given the negative impact of parasitism in animal welfare, animal health and their reproductive ability, the anthelmintic control is an important issue in breed recovery programs [10,11,12].

In the second year of the study, between December 2010 and September 2011, an increase in the annual prevalence (from 35.1% to 58.5%) was observed, with a maximum of 98.4% registered in March 2011. During the second year, an increase in the prevalence was observed in all the age groups. The weather characterization of the region showed a marked decrease in precipitation during this period. According to the records of the Monthly Climatological Bulletin of Continental Portugal from the National Institute of the Sea and Atmosphere (IPMA), 2011 was a dry year, with an average annual rainfall of only 59.5 mm. In that year, July was the driest month (1.1 mm) and September was the wettest (136.8 mm). Considering that the water lines of the region depend on the climatic conditions [33,54] and that the traditional method of irrigation used in pastures, which is a regulating factor in the cycle of water and in soil nutrients, can be interrupted in the driest years when the water courses dry completely [55,56]; it is clear that geoclimatic conditions are important to maintain the floristic composition of the spontaneous vegetation characteristic of pastures. Thus, water availability is a critical element for the existence of pasture [57]. Food scarcity can change the natural behavior of donkeys that start to feed nearby defecation areas, increasing the risk of reinfection and infection of other animals. Under these conditions, donkeys can be infected by large numbers of strongylids [58,59,60,61,62,63]. Moreover, the sharing of pastures promotes an increase of parasitism of the animals, with an increase in egg excretion [31]. In the studied region, pastures are used to feed ruminants and domestic equids and frequently ruminants and equids share pastures [54,57,64]. An infection by *Trichostrongylus axei* was observed in the second year of the study. It occurred in 0.8% of animals, namely, in a six-year-old castrated male. This finding suggests that joint grazing of donkeys and ruminants allowed the transmission of parasites that are commonly found in ruminants, as reported in other studies [65,66]. 

Regarding the internal factors that can influence TST, in this study, a higher prevalence was observed in females (39.5%) than in males (37.1%). Additionally, a higher prevalence was observed in animals younger than 4 years (46.7%) and a lower prevalence in animals older than 10 years (35.6%). Furthermore, a higher prevalence was observed in animals with a lower body condition (40.8%) than in those with higher body condition (35.8%). According to the results, the thoracic perimeter seems to have no relation to the infection prevalence rate. This result may be related to the morphometric uniformity observed in the studied population. Thus, it was possible to identify the sex, the age and the body condition index as additional indicator factors for TST. In fact, a higher intensity of strongylid parasitism was noted in females, younger than four years and showing a lower body condition, which agrees with reports from several other authors [67,68]. 

During the second and third years, an increase of parasitism was observed, which was associated with an increase of the prevalence and increase of egg output measured in females and in the youngest animals. Regarding sex, no significant differences were observed between the prevalence in females (39.5%) and in males (37.1%), as well as between the total value of EPG excreted by females (564.5 EPG) and males (520.3 EPG). However, the frequency of new infections was higher in females (6.9) than in males (2.6), as well as the excretion of eggs observed during the driest period of the study, in which females presented values of 779.9 EPG and 660.7 EPG, and males 554.5 EPG and 502.3 EPG, in the second and third years, respectively. In the group of animals younger than four years, a peak of excretion occurred in the second year (901.6 EPG) and third year (709.0 EPG). Overall, this study allowed an observation of a greater susceptibility of young animals, as well as females, to parasitism by strongylids, which is mentioned by other studies [69,70,71].

This study also allowed the development of an annual curve of excretion of strongylid eggs for Miranda do Douro donkeys living in the northeast of Portugal. This curve reflects a seasonal pattern of egg excretion, with a progressive increase from December to June, when a maximum peak in egg shedding is observed, thereafter decreasing excretion gradually from September to December. A seasonal pattern of excretion was also described in wild equids from the northwestern Portugal [31] and northwestern Spain [72] as well as in countries further north, such as the United Kingdom [73,74], Ukraine [75] and the United States [76]. The dynamics of strongylids, namely cyathostomins, depend on the environmental conditions that interfere with the egg-laying seasons. When environmental conditions are unfavorable, which in the northern hemisphere occurs from December to March, the cyathostomin larvae remain encapsulated in the mucosa and submucosa of the large intestine, suspending the endogenous development until adulthood. The low excretion of cyathostomins eggs observed from December to March, a period of extreme environmental conditions, unfavorable to exogenous development, seems to show the presence of poorly viable adult strongylids in the lumen of the large intestine [77]. In late winter or early spring, adult senescent strongylids are eliminated and hypobiotic larvae emerge into the lumen of the large intestine [78,79].

The annual excretion curve of strongylid eggs in donkeys from the Miranda do Douro breed in the northeast of Portugal corresponds to the annual cyathostomin population dynamics cycle described for horses in the northern hemisphere. It is influenced by meteorological conditions observed in the region. In this study, conditions seem to be favorable for the development of the exogenous strongylid cycle from March to June. First-stage larvae develop in a range of temperatures, from +3 °C to +40 °C, hatch and then survive for several weeks at low temperatures (+8 °C). In the northeast of Portugal, temperatures around +8 °C, thus favorable temperatures for the development of strongylid eggs, are observed from March. This occurrence may explain the increase of third-stage larvae in pastures in April, about one month after the increase of egg shedding (data not shown). The peak of this egg excretion is observed in June. During the warmest months (July and August), temperatures reach 40 °C, which are unfavorable conditions for the exogenous development of strongylids. Thus, a decrease in egg excretion is observed from July. Hence, the obtained results agree with the meteorological conditions described by several authors referring to the existence of a strong continental influence in this region in opposition to the Atlantic influence observed in the rest of the country. This continental influence is characterized by a wide range of temperatures: hot and short summers, and cold and long winters [33,54,80]. 

Parasite infections are common in donkeys. The prevalence of infections by intestinal strongylid in donkeys observed in this study is similar to results described in other studies ranging from 82.0 to 90.0% [19,81,82,83,84,85].

In this study, the TST lowered the biodiversity of gastrointestinal parasites. Cyathostomins were the most prevalent over the five years. This finding was also observed in other studies that showed a prevalence close to 100% [9,10,31,45,85,86,87,88,89] and a low biodiversity among the parasite’s population associated to the parasite control [25,46,48,90]. Similar results were found previously when a strategic control was implemented [29].

The cyathostomin infection observed in this study may represent a risk to animal health since these parasites may have a negative impact on health, causing a decrease in the body condition of infected animals [91,92,93], and possibly causing severe colitis, chronic diarrhea or mortality of up to 50% in equids [15,94]. Moreover, they may represent a challenge for antiparasitic control, as these parasites are associated with emerging anthelmintic resistance to all groups of synthetic compounds available for anthelmintic treatment of equids [25,48,90,95]. 

*Cyathostomum* sensu lato was the only genus of cyathostomins observed over the five years of this study. Morphotypes A (35.0 to 85.7%) and D (15.6 to 30.8%) were the most prevalent. However, during the second and the third years, an increase of the biodiversity of the genus *Cyathostomum* sensu lato was observed. This finding is similar to another study showing a high prevalence of *Cyathostomum* sensu lato in stabled donkeys and mules, and a high biodiversity of this genus in non-dewormed animals [96]. *Cyathostomum* type A was frequently observed, with an abundance of 65 to 98%, followed by morphotypes C and D, from 6 to 8%, and morphotypes B, E, F, G and H were less frequently observed. Antiparasitic control measures reduce the biodiversity of this genus [29,96].

*Strongylus vulgaris* was the most observed strongylin, showing a prevalence of 2.0% during second year of the study. Similar results are reported in other studies [88,89,97,98,99]. It occurred in one animal with no indication for treatment, and due to the identification of third-stage larvae of *Strongylus vulgaris*, an anthelmintic treatment was advised given the pathogenic importance of this agent [100,101,102]. The appearance of *Strongylus vulgaris* has been observed on farms with a reduction of anthelmintic treatments over several years. Thus, the role of TST in the increase in prevalence of *Strongylus vulgaris* has been suggested [25,45,46,103,104,105], but further studies are needed to assess this possibility [62,105].

*Parascaris equorum* was found, during the second year of study, in 0.8% of animals. Infections by this parasite have also been described in other studies [10,65,66,69,71,88]. This finding suggests the absence of protective immunity against infection by ascarids in adult donkeys [106,107].

Overall, the obtained results enhance knowledge regarding the effect of TST in the Miranda do Douro donkey breed population in rehabilitation centers from the northeastern region of Portugal. These results are very promising for developing an integrated and more rational parasite control in this autochthonous donkey breed.

## 5. Conclusions

This study allowed the characterization of the population of donkeys from the autochthonous Miranda do Douro donkey breed, their parasites, as well as the meteorological conditions of the region which influenced its prevalence and seasonality during the study period.

The results of the study clearly confirmed that the implementation of the TST approach allowed a more rational use of anthelmintics by limiting the number of treatments. Moreover, it promoted a decrease in the prevalence of parasitism.

Overall, this study showed: (i) A negative binomial type distribution of intestinal parasitic strongyles of donkeys, in which about 19.4% of donkeys were regularly dewormed, concentrate the totality of parasitic strongyles of donkeys; (ii) The influence of husbandry in parasitism, such as pastures, which are the basis for feeding ruminants and domestic equids in this region and may cause cross infections; (iii) The influence of the climatic seasonality in donkeys strongylidosis, expressed by the annual curve of excretion of strongylid eggs; (iv) The influence of internal factors that can act as indicator factors of parasitic infection by strongylids in this population; (v) A decrease in biodiversity of gastrointestinal parasites induced by TST, namely by using only ivermectin as a dewormer. 

The influence of multiple factors on parasitism is an important finding, suggesting that optimal treatment strategies for donkeys need to be tailored to climatic regions and herd structure and management.

## Figures and Tables

**Table 1:** Monthly average data on weather conditions in the region during the study period (2009–2014).

	Temperature (Mean) (°C)	Maximum Temperature (Mean) (°C)	Minimum Temperature (Mean) (°C)	Rainfall (Mean) (mm)
January	5.1	9.1	1.2	112.6
February	5.4	10.8	0.0	102.2
March	9.2	14.2	3.8	72.6
April	11.9	17.7	6.1	75.8
May	14.5	21.1	8.0	49.2
June	18.4	25.6	11.2	30.6
July	22.0	30.0	14.0	14.6
August	21.9	30.1	13.6	14.6
September	19.4	26.8	11.9	45.4
October	14.1	20.1	8.1	112.4
November	8.3	12.9	3.7	95.4
December	4.9	12.9	0.5	139.3

**Table 2:** Descriptive characteristics of the studied population ^a.^

	Females	Geldings
Age (years)	6.8 (SD = 5.2; Max = 20; Min = 2)	14.5 (SD = 6.3; Max = 20; Min = 4)
Body Condition Index	3.2 (SD = 0.7; Max = 5; Min = 2)	3.2 (SD = 0.5; Max = 4; Min = 2.5)
Thoracic Perimeter (cm)	141 (SD = 11.5; Max = 168; Min = 115)	142 (SD = 14.4; Max = 163; Min = 120)

^a^ data regarding the category of breeding males was not included as it consisted of only one animal.

**Table 3:** Summary of data considering categories of body condition index and thoracic perimeter per sex.

	Categories	Females (*n*)	Geldings (*n*)	Breeding Male (*n*)
Body Condition Index (groups)	2–2.5	9	3	-
	3–3.5	25	12	1
	4–5	8	14	-
Thoracic Perimeter (groups)	115–129	7	5	-
	130–149	25	7	1
	150–168	10	7	-

**Table 4:** Prevalence of parasitism observed in the population during the study period, per parameter.

		Prevalence (%)	Confidence Interval 95% (%)
Age group	Younger than 4 years	46.7	16.7–78.9
	4 to 10 years	38.2	21.8–57.6
	Older than 10 years	35.6	17.8–60.0
Sex	Female	39.5	27.1–55.5
	Geldings	37.1	19.2–59.0
Body Condition Index	2–2.5	40.8	16.0–70.7
	3–3.5	38.9	25.6–58.3
	4–5	35.8	12.8–66.7
Thoracic Perimeter	115–129	40.0	19.3–68.1
	130–149	40.0	24.7–56.3
	150–168	37.6	17.3–58.7

**Table 5:** Abundance of third-stage larvae of gastrointestinal parasites per month and per year of the study period.

	December	March	June	September	Total (year)	Average	Standard Deviation
1st year	2200	2000	2700	1600	8500	2125.0	457.3
2nd year	1400	6026	3695	3100	14,221	3555.3	1912.8
3rd year	2803	2710	3400	1900	10,813	2703.3	616.6
4th year	1500	2200	2700	1700	8100	2025.0	537.7
5th year	1400	1401	1700	1200	5701	1425.3	206.1
Total (month)	9303	14,337	14,195	9500	47,335	n.a.	n.a.
Average	1860.6	2867.4	2839.0	1900.0	9467.0	n.a.	n.a.
Standard Deviation	624.0	1826.9	771.9	717.6	3217.1	n.a.	n.a.

n.a. Not applicable.

**Table 6:** Abundance of strongylid third-stage larvae per month over the study period.

L3 *n* (%)		Cyathostominae %								Strongylinae %	
		Genus *Cyathostomum* Morphotypes									
		A	B	C	D	E	F	G	H	*S. vulgaris*	*Oesophagodontus*
December	9303 (19.7)	91.22	0.08	1.34	7.14	0.01	0.01	0.20	0.00	0.00	0.00
March	14,337 (30.3)	91.50	0.13	1.40	6.03	0.03	0.04	0.38	0.00	0.04	0.01
June	14,195 (30.0)	90.98	0.09	1.20	7.09	0.02	0.03	0.22	0.01	0.02	0.00
September	9500 (20.0)	93.56	0.08	1.11	5.04	0.02	0.02	0.15	0.00	0.02	0.00
Total	47,335	91.7	0.1	1.3	6.4	0.02	0.03	0.3	0.002	0.02	0.002

## Data Availability

The data presented in this study are available upon request from the corresponding author.
